# Are neurodegenerative diseases late-onset neurodevelopmental disorders? Tracing the developmental origins of neuronal vulnerability

**DOI:** 10.3389/fnins.2026.1729102

**Published:** 2026-02-24

**Authors:** Julian Cheron, Adrian Ranga, Jerome Bonnefont

**Affiliations:** 1 Department of Biomedical Engineering, Johns Hopkins University, Baltimore, MD, United States; 2 Laboratory of Bioengineering and Morphogenesis, Department of Mechanical Engineering, KU Leuven, Leuven, Belgium; 3 Brain Evo-Devo and Disease, Department of Neurosciences, Faculty of Medicine, Pharmacy and Biomedical Sciences, University of Mons, Mons, Belgium; 4 Faculty of Medicine, Université Libre de Bruxelles, Brussels, Belgium

**Keywords:** brain development, brain organoids, developmental misprogramming, human brain evolution, neurodegenerative diseases, selective neuronal vulnerability

## Abstract

Neurodegenerative diseases are traditionally viewed as age-associated conditions, characterized by distinct biochemical, cellular, and clinical features. However, emerging evidence suggests that their origins may trace back to much earlier stages of life. In this review, we synthesize insights from molecular genetics, developmental neurobiology, and systems neuroscience to examine the hypothesis that selective neuronal vulnerability can arise from developmental misprogramming. We explore how early-life processes—ranging from neurogenesis to synaptic maturation and circuit formation—can imprint long-lasting susceptibilities that manifest as degeneration decades later. Crucially, we highlight that many neurological disorders share early developmental commonalities that may predispose individuals to neurodegenerative vulnerability later in life. This is most apparent in familial forms of these diseases but may also emerge through embryonic or perinatal interactions with environmental or polygenic risk factors. Furthermore, we emphasize the importance of human-specific developmental features, which not only advance our understanding of brain formation but also reveal unique vulnerabilities to neurodegenerative diseases—insights that are increasingly accessible through advances in 3D organoid modeling. Together, these perspectives support a conceptual reframing of neurodegeneration as a late-onset neurodevelopmental disorder. This shift opens promising avenues for early diagnosis, prevention, and precision therapeutics, redirecting focus from late-stage intervention to fostering developmental resilience.

## Introduction

Neurodegenerative diseases encompass a wide and complex spectrum of disorders clinically manifesting through multiple symptoms, including cognitive decline, motor impairments, and behavioral or psychiatric disturbances, depending on the specific neuronal populations initially affected and the disease’s trajectory. Mostly described between the 19th century —such as Parkinson’s disease, amyotrophic lateral sclerosis, Huntington’s disease, or Friedreich’s ataxia—, and the early 20th century with conditions like Alzheimer’s, Pick’s, and Creutzfeldt-Jakob diseases, these disorders have now evolved into a major global health and socioeconomic challenge. This burden has been significantly intensified by population growth and aging demographics, with recent estimates indicating that over 3.4 billion individuals are affected, representing more than 40% of the worldwide population, with a surge in prevalence of nearly 20% in the past three decades (Steinmetz et al., 2024).

Despite years of research, the initial mechanisms that trigger neuronal degeneration remain poorly understood. A deeper understanding of these early pathophysiological events is essential to identify novel diagnostic tools and therapeutic targets, design multimodal therapies capable of correcting the diverse molecular, biochemical, and cellular disruptions observed across neurodegenerative conditions, and to rationally develop gene or cell-replacement therapies and precision medicine approaches.

While knowledge of these diseases has expanded considerably over recent decades-largely emphasizing their distinct molecular and clinical features-a growing body of literature now suggests that their origins may lie much earlier than previously assumed, potentially rooted in shared developmental pathways. This review therefore highlights developmentally “hidden” alterations that may predispose individuals to neurological diseases, but which remain compensated until genetic risk factors or environmental exposures come into play, disrupting protective mechanisms and precipitating the molecular onset of disease in adulthood.

## Biochemical and cellular pathophysiological components of neurodegenerative diseases

Neurodegenerative diseases exhibit considerable heterogeneity in terms of neuronal populations they affect, first inducing localized neurodegeneration in specific regions, such as the cerebral cortex, hippocampus, cerebellum, or spinal cord. As these diseases progress, pathological mechanisms often propagate across other parts of the nervous system, ultimately causing the death of multiple neuronal populations and the emergence of clinical symptoms that may appear decades after the initial molecular insult (Braak et al., 2003; McCann et al., 2016).

Despite this diversity, neurodegeneration can be defined by three progressive stages: (i) an initial trigger of biochemical nature, (ii) a cellular response, and (iii) a dysregulation of brain homeostasis, culminating in structural degeneration and functional decline (Balusu et al., 2023).

One of the most frequently occurring biochemical triggers of neurodegeneration involves the initiation of aberrant changes in protein conformations, resulting in toxic aggregations. Such protein aggregates are formed by *β*-sheet-rich protein structures, which are more resistant to ubiquitin-mediated proteasomal degradation. Historically, pathological changes in protein conformation were attributed to mutations in familial forms of neurodegenerative diseases (Hardy and Selkoe, 2002; Rosen et al., 1993) or to altered processing that favored pathogenic isoforms over physiologically neuroprotective ones (Cuervo et al., 2004; Furukawa et al., 2006; Iqbal et al., 2005; Mawuenyega et al., 2010; Neumann et al., 2006). More recently, it has been recognized that multiple proteins, including Aβ, Tau, *α*-synuclein, TDP-43, FUS, and SOD1, can engage in propagation mechanisms similar to those in prion diseases, where, even in the absence of genetic mutations, they can spontaneously misfold, self-template, and spread across synaptically connected regions (Jucker and Walker, 2018; Prusiner, 2012).

Heterozygous mutations in genes linked to dominantly inherited neurodegenerative diseases can also drive pathology through a toxic gain of function —via the accumulation of aberrant RNA and/or proteins— combined with a loss of physiological function due to haploinsufficiency. This dual mechanism is exemplified by HTT in Huntington’s disease (Laundos et al., 2023; Schulte and Littleton, 2011) and C9ORF72, which is implicated equally in amyotrophic lateral sclerosis (ALS) and frontotemporal dementia (FTD) (Braems et al., 2020). In contrast, Friedreich’s ataxia is a more classical loss-of-function disorder, caused by *FXN* gene silencing -primarily through epigenetic and transcriptional mechanisms- resulting in Frataxin deficiency (Al-Mahdawi et al., 2008; Li et al., 2015; Rai et al., 2008).

Adding further complexity, mixed proteinopathies are increasingly recognized. For instance, Tau aggregates, historically considered as a major driver of Alzheimer’s disease and primary tauopathies, have now been identified in *α*-synuclein-positive Parkinson’s disease brains (Chu et al., 2024; Zhang et al., 2018). These proteins may cross-seed, directly interacting to promote each other’s aggregation or enhancing the hyperphosphorylation of the other through signaling pathway crosstalks (Credle et al., 2015;^,^ Pan et al., 2022), thereby complicating the clinical picture by blending multiple symptoms (Wingo et al., 2022).

These biochemical alterations converge to ultimately disrupt several molecular systems, including proteasomal and autophagic degradation pathways, synaptic transmission, and organelle dysfunction, particularly the endoplasmic reticulum, the Golgi apparatus, and mitochondria. Mitochondria dysfunction is especially critical, ranging from impaired mitophagy to decreased biogenesis, leading to oxidative stress and metabolic imbalance (Wong and Krainc, 2017). Despite these shared features, each neurodegenerative disease retains distinct molecular signatures. For example, Tau pathology destabilizes axonal microtubule (Best et al., 2019; Black et al., 1996; Jinwal et al., 2010), ɑ-synucleinopathy disrupts the SNARE complex assembly, impairing synaptic vesicle fusion and transmission (Wong and Krainc, 2017), and *Huntingtin* mutation compromises neuronal survival by impairing BDNF trophic support at multiple levels (Nucifora et al., 2001; Saudou and Humbert, 2016).

While neurons are the primary targets of neurodegenerative diseases, the molecular and biochemical disruptions extend beyond them, affecting other non-neuronal brain cells and marking the transition to the “cellular phase” of neurodegenerative disease progression (Balusu et al., 2023). Consequently, astrogliosis, microgliosis, changes in vasculature, and activation of immune and inflammatory pathways will be broadly observed across neurodegenerative conditions. Nonetheless, disease-specific mechanisms persist, such as gene expression profiles leading to distinct disease-associated markers and processes in these non-neuronal cells (Balusu et al., 2023), and risk-associated polymorphisms. For instance, genome-wide association studies in Alzheimer’s disease have identified numerous risk variants enriched in non-neuronal cell types (Kunkle et al., 2019), whereas risk polymorphisms are restricted to neurons in others, like Parkinson’s disease (Nalls et al., 2019). While these structured polygenic risk landscapes act primarily as modifiers of disease susceptibility rather than as direct etiological determinants, they nonetheless challenge the earlier perception that non-familial neurodegenerative disorders are primarily sporadic. Instead, they suggest that such conditions represent non-deterministic polygenic states with incomplete penetrance of genetic risk, in which aging and environmental factors will jointly determine a potential disease emergence later in life (Arendt et al., 2017).

This shift in perspective also broadens the scope of pathophysiological inquiry. For instance, *GPNMB* variants have been identified in Parkinson’s patients where the encoded Glycoprotein nmb interacts with neuronal ɑ-synuclein (Balusu et al., 2023; Diaz-Ortiz et al., 2022), while polymorphisms in *TREM2*, *CR1*, or *SORL1,* encoding receptors expressed in microglia, favor Alzheimer’s disease (Balusu et al., 2023; Kunkle et al., 2019). But polymorphisms are not always required for a gene to be implicated in the pathophysiology of the neurodegenerative diseases, as in the example of *GPNMB*. This gene shows no polymorphisms neither in Alzheimer’s disease, nor in Nasu-Hakola disease, a rare autosomal recessive disorder characterized by progressive presenile dementia, despite GPNMB protein levels accumulate in subsets of microglia of these patients as a part of their activation state, suggesting that cell-type-specific gene expression dynamics, rather than genetic variation alone, may also drive pathogenic roles (Cheron et al., 2023; Hüttenrauch et al., 2018; Satoh et al., 2019).

These insights raise fundamental and still unresolved questions: what triggers the high degree of specificity in the initial vulnerability of brain regions in each neurodegenerative disease, in which cell type, and how this leads to selective neuronal subtype sensitivity during the initial biochemical phase, given the multiple number of gene risk factors, their expression pattern, and their specific association to a symptom-related neurodegenerative spatiotemporal pattern? Paradoxically, despite the emphasis on specific expression pattern of risk-associated genes, these puzzling interrogations are also illustrated by the counter-example that the causatives genes of the familial forms of these neurodegenerative diseases, like *APP* and *PSEN1/2* (Alzheimer’s)*, MAPT* (frontotemporal dementia), *SNCA* and *LRRK2* (Parkinson’s), *SOD1* (amyotrophic lateral sclerosis), and *FXN* (Friedreich’s Ataxia) are expressed throughout the brain and involved in fundamental cellular processes (Shabani et al., 2023). Yet, certain brain regions and neuronal subtypes will specifically succumb early in each disorder, while others will remain resilient.

Another complicating factor is that clinical heterogeneity —both in terms of onset and symptomatology— is observed among individuals with the same diagnosis during the early stages of these diseases. Recent positron emission tomography imaging studies associated with progressive patterns of clinical decline described that distinct Alzheimer’s or Parkinson’s disease subtypes exist and are associated with multiple trajectories of Tau or ɑ-synuclein spatiotemporal spreading, respectively (Vogel et al., 2021; Zhou et al., 2023). For instance, in Alzheimer’s disease, initial Tau accumulation in limbic areas is linked to carrying *APOEƐ4* allele, a late onset of the disease and prominent memory deficits, mirroring Braak stages of parenchymal Aß plaque deposition hierarchy (Thal et al., 2008). However, three other Tau trajectories have been reported, each associated with a specific clinical profile. When abnormally high Tau levels arise in the cerebral cortex, including the sensory regions, this leads to an early Alzheimer’s disease onset, sensory dysfunction and relatively preserved memory; neurofibrillary tangle accumulation originating in the visual cortex leads to visuospatial impairments and slower disease progression; and asymmetrical Tau aggregation in the left cortical hemisphere elicits language deficits associated with rapid disease progression (Vogel et al., 2021).

## Origin of selective vulnerability in mature neurons

The precise sources of selective neuronal vulnerability in neurodegenerative diseases remain incompletely understood and is likely multifactorial. One hypothesis posits that decreased cellular fitness in vulnerable neurons is linked to highly specific gene expression profiles. For instance, the neurofilament (NF)-triplet, comprising three genetically and structurally interrelated NF-light, NF-medium, and NF-heavy subunits, is co-expressed in certain neuronal populations to form intermediate filaments. Notably, layer III and V pyramidal neuron subpopulations, which are particularly susceptible to Tau pathology, express this NF-triplet. A similar vulnerability is observed in the GABAergic interneurons that also express the NF-triplet, in contrast to the NF-triplet-negative neurons, which typically do not exhibit neurofibrillatory tangles in Alzheimer’s patient brains (Mitew et al., 2013; Sampson et al., 1997). More recently, large-scale single-nuclei transcriptomic profiling of post-mortem prefrontal cortex samples from individuals at various stages of Alzheimer’s disease revealed that distinct excitatory and inhibitory neuronal subpopulations exhibit early, pathology-associated transcriptional changes well before the onset of global stress responses (Mathys et al., 2019). However, establishing causal links between gene expression patterns and neuronal vulnerability remains a significant challenge.

Functional characteristics of specific neuronal subpopulations may also contribute to their selective vulnerability (Papanikolaou et al., 2025). A common feature across neurodegenerative diseases is that the most affected neurons exhibit tightly regulated excitability, rendering them particularly sensitive to disruptions in calcium homeostasis and excitability loss (Roselli and Caroni, 2015). Differences in intrinsic stressor thresholds further modulates neuronal vulnerability (Saxena and Caroni, 2011). Moreover, neurons with high metabolic demands may be disproportionally affected by global biochemical changes (Roselli and Caroni, 2015). Consistently, early quantitative analyses showed that action potentials and synaptic transmission impose substantial energy costs, with even modest increases in firing rate leading to sharp elevations in metabolic load (Attwell and Laughlin, 2001).

In this context, midbrain dopaminergic neurons —characterized by elevated mitochondrial bioenergetics and extensive axonal arborization which heightens their metabolic burden— are particularly vulnerable to degeneration (Pacelli et al., 2015). Computational modeling further supports this view, showing that reduced ATP availability in motor neurons can trigger prolonged depolarization, massive Ca^2+^ influx, and ultimately cell death (Le Masson et al., 2014). This model revealed a self-reinforcing loop in which minor ATP deficits lead to ionic imbalances, increasing energy demand and exacerbating the energy shortfall. Importantly, even localized energy deficits at axon terminals can initiate retrograde degeneration affecting the entire neuron. This framework helps also explain the differential vulnerability of slow versus fast-fatigable motor neurons in amyotrophic lateral sclerosis, with the latter succumbing under milder bioenergetic stress (Johri and Beal, 2012; Schon and Przedborski, 2011).

## Hypothesis of a neurodevelopmental origin of vulnerability

Beyond electrophysiological traits, neuronal vulnerability may also emerge from network-level mechanisms shaped by the brain’s intricate connectivity (Boulanger and Shatz, 2004). Functional magnetic resonance imaging and atrophy mapping studies have shown that distinct neurodegenerative syndromes target specific intrinsic connectivity networks, suggesting that regional vulnerability is more influenced by connectivity patterns than by anatomical cell proximity (Seeley et al., 2009). This is particularly relevant in prion-like disease, where pathological proteins propagate along these networks (Gousset et al., 2009; Kim et al., 2019). Supporting this, computational models simulating the diffusion of misfolded proteins along the brain’s structural connectome have successfully reproduced the characteristic atrophy patterns seen in Alzheimer’s disease and frontotemporal dementia (Raj et al., 2012).

Importantly, the foundational architecture of these networks is established prenatally and refined throughout early life (Sherwood and Gómez-Robles, 2017). This raises the possibility that miswiring mechanisms with developmental origin may lay the ground for later vulnerability. Initially proposed in the context of psychiatric diseases as they are more prone to develop at a pediatric age (Chini et al., 2020; Di Martino et al., 2014; Hansen et al., 2022), this concept is now being extended to neurodegenerative diseases, where hallmark proteins are known to play physiological roles in synaptic transmission and plasticity. For instance, newborns exhibit high levels of Tau phosphorylation which promotes axon remodeling and synaptic plasticity (Arendt, 2004; Mattsson et al., 2010). In adulthood, the persistence of such developmental phosphorylation programs may however become detrimental and create molecular contexts that interact with disease-specific processes: in Alzheimer’s disease, some brain regions that mature more slowly or retain remodeling capacities in adulthood might be predisposed to neurofibrillatory tangle formation and Alzheimer’s pathology (Arendt et al., 1998; Moceri et al., 2000). Amyotrophic lateral sclerosis and frontotemporal dementia may also originate from early circuit alterations as observed following synapse-disrupting *SOD1* and *C9ORF72* mutations (Hendricks et al., 2023; Martin et al., 2013; Van Der Geest et al., 2024). Loss of Huntingtin in postmitotic neurons transiently disrupts glutamatergic synapse maturation and transmission during perinatal circuitry, a defect that only manifests as Huntington’s pathology later in life (Braz et al., 2022; Wennagel et al., 2022). These findings suggest that developmental circuit assembly may imprint long-lasting “at-risk” states on specific neuronal populations (Shabani and Hassan, 2023).

It is now proposed that neurodegenerative diseases could have an additional, and even earlier, developmental origin, during neurogenesis itself. Divergent developmental trajectories between neuronal subtypes could render some populations intrinsically more susceptible to later-life cellular stressors, reframing some, if not all, these conditions as “late-onset neurodevelopmental disorders” (Shabani and Hassan, 2023).

Supporting this, in the context of non-familial neurodegenerative diseases which represent by far the most prevalent form of these disorders, numerous studies exposing rodents or non-human primates to in utero or perinatal environmental toxicity has been shown to result in delayed specific neurodegenerative phenotypes emerging in adulthood (reviewed in Gauvrit et al., 2022; Modgil et al., 2014; Tartaglione et al., 2015), in line with long-term consequences of early toxic insults in human (Borenstein et al., 2006; Gauvrit et al., 2022; Logroscino, 2005). Consistent with this view, models of maternal immune activation also indicated that early-life brain inflammation can enhance adult vulnerability to elicit subsequent environmental toxins and neurodegenerative-like phenotypes (Tartaglione et al., 2015).

Epigenetic regulation has emerged as a central interface between genetic predisposition, environmental exposure, and long-term neuronal vulnerability (e.g., Kochmanski et al., 2024). Environmental factors—including metals, pesticides, air pollution, or dietary imbalance—can durably reshape gene-regulatory landscapes through epigenetic mechanisms, thereby increasing disease risk and modulating selective neuronal vulnerability (Migliore and Coppedè, 2009). Recently, adult somatic cells from patients with non-familial Alzheimer’s disease have been used to examine how epigenomic memory retained during hiPSC reprogramming may inform the developmental origin of neurodegenerative diseases. When differentiated into cortical organoids, these hiPSCs spontaneously developed Aβ and Tau pathologies, indicating that non-genetic, developmentally imprinted alterations may predispose neurons to late-onset disease phenotypes (Katbe et al., 2026).

**Figure 1:**
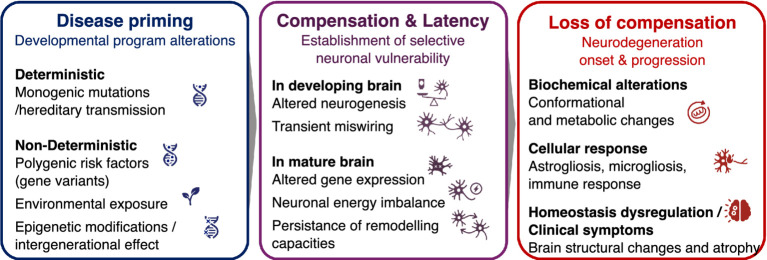
Developmental framework of neurodegenerative diseases. Neurodegenerative diseases may originate from early-life priming during embryonic or perinatal developmental stages, driven either by disease-causing familial mutations or by a non-deterministic interplay of environmental factors (e.g., metals, pesticides, maternal dietary imbalance), polygenic risk factors with incomplete penetrance, and epigenetic modifications (left). These factors can induce subtle alterations in brain development, establishing neuronal vulnerability – either independently or alongside changes in the mature brain that reflect or further contribute to vulnerability acquisition. Importantly, this vulnerability does not necessarily lead to disease onset, as compensatory mechanisms can preserve neural function for extended periods (middle). With aging, cumulative life stressors such as environmental challenges or additional epigenetic changes may erode these compensatory processes, triggering neurodegeneration in individuals with non-deterministic risk factors, whereas familial mutations typically result in earlier disease onset (right). See Figure 2 for individual trajectories

Early environmental exposures may therefore establish latent, compensated vulnerabilities that remain silent for decades but can interact with adult environmental exposures, genetic background and aging-related processes to eventually precipitate neurodegeneration in some individuals. Moreover, environmentally driven epigenetic modifications raise the possibility of intergenerational effects (Seto et al., 2024)—and potentially transgenerational priming of vulnerability (Takahashi et al., 2023)—that may further bias developmental trajectories without constituting deterministic inheritance (Figure 1).

While animal models have been instrumental in advancing our understanding of the molecular mechanism of neurodegenerative diseases and remain essential for probing circuit-level and systemic mechanisms (Dawson et al., 2018; Kim et al., 2019), *in vitro* models based on human induced pluripotent stem cells (hiPSCs) carrying disease-associated mutations —whether introduced genetically or derived from patients— now enable to directly and mechanistically explore how altered developmental trajectories may contribute to the origins of neurodegenerative diseases, when layers of *in vivo* regulation are removed in the minimal *in vitro* environment.

Exemplifying this, hiPSCs carrying Alzheimer’s disease-linked mutations, such as *APP* knockout and *PSEN1* variants exhibit premature neurogenesis (Arber et al., 2021; Shabani et al., 2023). While the precise role of APP remains unclear, notably whether its loss phenocopies its mutation or reflects a broader developmental function, Presenillins, as components of the *γ*-secretase complex, likely influence both Notch receptor activation and APP processing into Aß, thereby regulating multiple aspects of neuronal development and maturation (Selkoe and Kopan, 2003; Zhang et al., 2020). A similar acceleration in cell-cycle exit has also been observed during the cortical differentiation of hiPSCs with a repeat expansion in ALS/FTD-associated *C9ORF72* (Hendricks et al., 2023), as well as during the dopaminergic differentiation of hiPSCs carrying a *LRRK2*-*G2019S* mutation (Walter et al., 2021), the most prevalent monogenic cause of Parkinson’s disease (Kmiecik et al., 2024). Intriguingly, *LRRK2*-*G2019S* mutated human neural precursors also present pivotal mitochondrial defects previously described only in Parkinson’s disease-affected dopaminergic neurons (Walter et al., 2019), supporting the importance of mitochondrial behavior for normal neurogenesis (Iwata et al., 2020) and that their developmental defects may contribute to later manifestation of Parkinson’s pathology. In mouse models, *Huntingtin^Q111/Q111^* mutation also elicits premature neurogenesis during embryonic cortical development by altering spindle orientation and consequently the mitotic cleavage plane (Molina-Calavita et al., 2014). Similar effects are seen with *Huntingtin* silencing, indicating that polyQ expansion in *HTT* disrupts its developmental role (Godin et al., 2010).

Clinical observations further support this developmental hypothesis. Juvenile-onset forms of Parkinson’s and Huntington’s diseases have been documented (Shabani and Hassan, 2023). Functional magnetic resonance imaging studies in pediatric Friedreich’s Ataxia patients revealed spinal cord hypoplasia before overt neurodegeneration (Rezende et al., 2019), and postmortem studies confirmed that the spinal cords of Friedreich Ataxia’s patients reach a stable developmental plateau that however falls short of normal adult size (Koeppen et al., 2017).

**Figure 2:**
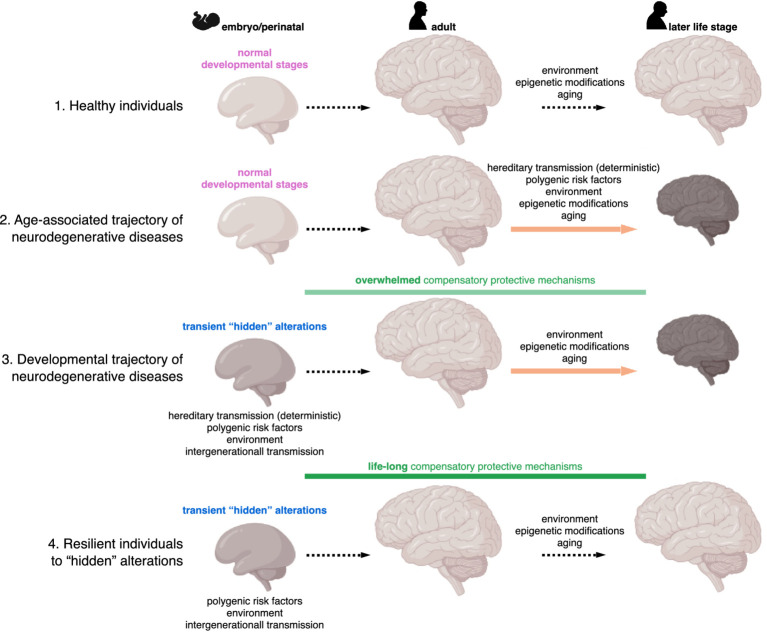
Individual models of neurodegenerative disease trajectories across the lifespan. Unlike healthy individuals (1), some develop neurodegenerative diseases, traditionally thought to result from neuronal vulnerability in adulthood, triggered by familial mutations or a combination of aging-related factors (2). The developmental framework proposes that familial mutations or a combination of factors may subtly impair brain development, initially compensated by protective mechanisms. Over time, these compensatory processes may become overwhelmed in some individuals (or inevitably fail in familial forms), leading to neuronal vulnerability and disease onset (3). However, others exhibit lifelong resilience, maintaining compensation that prevents neurodegeneration despite early disease priming (4).

Interestingly, not all individuals carrying risk-associated genes develop neurodegenerative diseases, raising the question of whether compensatory developmental mechanisms are sufficient to protect brain function throughout life (Figure 2). In others, these mechanisms may only delay onset until adulthood. Alzheimer’s disease, for example, has not been described in childhood or adolescence, implying long-term protective programs. In contrast, Sanfilippo syndrome, a childhood-onset dementia with Aß and Tau protein inclusions and transcriptomics and proteomics similarities to Alzheimer’s disease (Barthelson et al., 2025; Wiśniewska et al., 2024), manifest early as the implicated gene mutations cause heparan sulfate accumulation within lysosomes that overwhelms compensatory capacity (Benetó et al., 2020).

## Better understanding of developmental aspects for better understanding of neurodegeneration

This developmental framework not only reshapes our understanding of when and how neurodegenerative diseases begin but also invites a broader evolutionary perspective. A growing body of evidence suggests that the evolutionary expansion of the human cerebral cortex is intimately linked to our species-specific vulnerability to neurodegenerative diseases, particularly those that initiate pathology in cortical structures. For example, Alzheimer’s disease, which strongly affects the neocortex, is virtually absent in non-human species, whereas Parkinson’s disease, which targets the evolutionarily older basal ganglia, has been observed in other mammals, including non-human primates (Espuny-Camacho et al., 2017; Li et al., 2021). Strikingly, the brain areas that show the highest vulnerability to Tau pathology are also those that have undergone the greatest evolutionary expansion (Arendt et al., 2017).

Thus, the evolutionary innovations that distinguish the human brain and enabled advanced cognition may have introduced unique susceptibilities to late-onset neurodegeneration. To fully grasp the origins of these diseases, it is therefore crucial to comprehend how human-specific developmental programs and evolutionary pressures have shaped the architecture —and fragility— of the brain.

At the cellular level, this vulnerability is mirrored in the unique dynamics of human cortical neurogenesis. Unlike in rodents, where neurogenesis is rapid and temporally constrained, human cortical progenitors exhibit protracted self-renewal, allowing more rounds of divisions (Libé-Philippot and Vanderhaeghen, 2021). This increase in the absolute number of progenitors translates into a greater total output of neurons that is crucial for building a larger and more complex cortex, but that may also increase the risk of developmental perturbations that later manifest as neurodegeneration. Supporting this, in contrast to the observations during human neurogenesis mentioned above, neurogenesis is not affected by *APP* ablation during the faster murine cortical development (Shabani et al., 2023).

Moreover, cortical expansion in humans is not solely due to increased neuronal output, but also to the emergence of species-specific genes that regulate novel developmental mechanisms (Bonnefont et al., 2011; Bonnefont et al., 2008; Charrier et al., 2012; Libé-Philippot et al., 2023; Schmidt et al., 2021; Suzuki et al., 2018). These genes not only influence the diversity of cortical progenitors, but also their proliferative dynamics and lineage potential (Andrews et al., 2020; Cadwell et al., 2019; Iwata et al., 2020; Lui et al., 2011; Suzuki et al., 2018; Van Heurck et al., 2023).

To study these uniquely human processes, organoid technology has become an indispensable tool as brain organoids derived from human pluripotent stem cells recapitulate key aspects of cortical development and evolution, thereby offering a platform to investigate how developmental misprogramming contributes to selective neuronal vulnerability (Lancaster et al., 2013; Pașca et al., 2025; Van Heurck et al., 2023). For example, three-dimensional co-culture of *APP* mutation-carrying human neurons and astrocytes, together with microglia, exhibit hallmark features of Alzheimer’s disease: Aß inclusion, Tau hyperphosphorylation and aggregation, neuroinflammatory responses coupled to astrogliosis and microgliosis, and neuronal death. In contrast, these features are absent in 2D monolayer cultures, where only Aß aggregates are observed (Park et al., 2018). This highlights the importance of spatial dimensionality and cellular context in modeling the full cascade of neurodegenerative pathology (Abdel Fattah et al., 2023; Karzbrun et al., 2021). Human brain organoids has also been employed to model Huntington’s disease, where the introduction of expanded 70Q repeats in the *HTT* gene disrupts neural progenitor organization with an early integrated-stress-response signature linking mutant *HTT* to a progressive developmental metabolic failure preceding neuronal loss (Lisowski et al., 2024). Organoid models of ALS/FTD-associated *C9ORF72* demonstrate that repeat expansions similarly alter the distribution of neural progenitors, reduce the number of deep layer cortical neurons and affect synapse structure, again implicating developmental misprogramming as a potential axis of disease vulnerability (Van Der Geest et al., 2024). Beyond genetic models, organoids also enable to experimentally deconvolve age and microenvironmental exposures. For example, vascularized neuroimmune organoids exposed to brain extracts from sporadic-AD patients develop Aβ- and Tau-like aggregates, neuroinflammation, and synaptic loss within weeks, illustrating how an adult pathological milieu can precipitate degeneration on a developmentally primed neural substrate (Ji et al., 2025). Together these examples highlight two complementary developmental mechanisms revealed by organoid systems: (1) disease-causing alleles perturb progenitor behavior, metabolism, or glial maturation during defined neurogenic windows -often within the first weeks to months of differentiation, and (2) those perturbed developmental programs lower the threshold for subsequent age-related or environmental insults to elicit the hallmarks of neurodegeneration. Organoids therefore offer longitudinal access —both spatially across brain regions and temporally across developmental stages— to map when and how developmental events convert into lifelong vulnerability, and to identify early, mechanistically tractable intervention points.

## From developmental insight to translational innovation: reimagining therapeutic design

The hypothesis that neurodegenerative diseases may originate from human-specific developmental programs shaping selective neuronal vulnerability has the potential to reframe therapeutic strategies, shifting attention from irreversible late-stage neuronal loss toward early developmental misprogramming. This perspective opens conceptual avenues for interventions aimed at preserving circuit integrity and cellular resilience before overt degeneration emerges (Braz et al., 2022).

However, the translational relevance of such an approach is inherently constrained, as preventive or presymptomatic interventions would primarily apply to individuals carrying identifiable genetic risk factors, representing only a fraction of the population affected by neurodegenerative diseases. Within this context, the developmental framework underscores the urgent need for earlier diagnostic strategies capable of detecting subtle molecular or cellular alterations long before symptom onset, as exemplified by neonatal genetic screening of *SMN1/SMN2* copy number in spinal muscular atrophy (Varone et al., 2025). In contrast, validated biomarkers enabling early identification or stratification of sporadic neurodegenerative disorders based on developmental alterations remain largely unavailable. The concept of developmental vulnerability should therefore be regarded as a guiding research framework rather than an immediately actionable clinical paradigm. Longitudinal, population-based studies integrating genetic, environmental, intergenerational priming (Seto et al., 2024), and developmental variables will be essential to identify such biomarkers and to determine their predictive value.

Emerging therapeutic modalities—including antisense oligonucleotides, gene replacement strategies, and CRISPR-based genome editing—nonetheless begin to exemplify the clinical relevance of this shift (Nuñez et al., 2021), having demonstrated efficacy in presymptomatic or early-stage intervention for select monogenic neurodegenerative disorders, such as spinal muscular atrophy and amyotrophic lateral sclerosis (Benatar et al., 2022; Gagliardi et al., 2024), and showing promise in modifying early disease trajectories in Huntington’s disease (Dolgin, 2025).

Collectively, these advances lay the foundation for a biology-driven therapeutic paradigm that acknowledges the genetic and developmental contributions to neurodegeneration, while also highlighting its current scope and limitations. Rather than constituting a universal solution, this framework points toward earlier, more precise interventions for defined patient subsets, guided by emerging biomarkers and aligned with individual developmental risk trajectories.
